# Spatiotemporal and Socioeconomic Risk Factors for Dengue at the Province Level in Vietnam, 2013–2015: Clustering Analysis and Regression Model

**DOI:** 10.3390/tropicalmed5020081

**Published:** 2020-05-19

**Authors:** Polly Ashmore, Johanna F. Lindahl, Felipe J. Colón-González, Vu Sinh Nam, Dang Quang Tan, Graham F. Medley

**Affiliations:** 1 Department of Infectious Disease Epidemiology, London School of Hygiene & Tropical Medicine, London WC1H 9SH, UK; Felipe.Colon@lshtm.ac.uk; 2 Department of Medical Biochemistry and Microbiology, Uppsala University, SE-751 23 Uppsala, Sweden; johanna.lindahl@imbim.uu.se; 3 International Livestock Research Institute, Hanoi 10 000, Vietnam; 4 Department of Clinical Sciences, Swedish University of Agricultural Sciences, SE-750 07 Uppsala, Sweden; 5 National Institute of Hygiene and Epidemiology, Hanoi 10 000, Vietnam; VSN@nihe.org.vn; 6 General Department of Preventive Medicine, Ministry of Health of Vietnam, Hanoi 10 000, Vietnam; dangquangtan@yahoo.com; 7 Department of Global Health and Development, London School of Hygiene & Tropical Medicine, London WC1H 9SH, UK; Graham.Medley@lshtm.ac.uk

**Keywords:** vector-borne disease, arbovirus, dengue fever, Vietnam, spatiotemporal, socioeconomic, province

## Abstract

Dengue is a serious infectious disease threat in Vietnam, but its spatiotemporal and socioeconomic risk factors are not currently well understood at the province level across the country and on a multiannual scale. We explore spatial trends, clusters and outliers in dengue case counts at the province level from 2011–2015 and use this to extract spatiotemporal variables for regression analysis of the association between dengue case counts and selected spatiotemporal and socioeconomic variables from 2013–2015. Dengue in Vietnam follows anticipated spatial trends, with a potential two-year cycle of high-high clusters in some southern provinces. Small but significant associations are observed between dengue case counts and mobility, population density, a province’s dengue rates the previous year, and average dengue rates two years previous in first and second order contiguous neighbours. Significant associations were not found between dengue case counts and housing pressure, access to electricity, clinician density, province-adjusted poverty rate, percentage of children below one vaccinated, or percentage of population in urban settings. These findings challenge assumptions about socioeconomic and spatiotemporal risk factors for dengue, and support national prevention targeting in Vietnam at the province level. They may also be of wider relevance for the study of other arboviruses, including Japanese encephalitis, Zika, and Chikungunya.

## 1. Introduction

Dengue is an arbovirus spread by *Aedes aegypti* and *Aedes albopictus* mosquitoes. It has varied clinical manifestations, from mild fever to Dengue Shock Syndrome or Dengue Heamorrhagic Fever. It represents a major infectious disease threat in Vietnam with almost 184,000 cases reported in 2017 alone [1]. Cases are unevenly distributed across the country’s 63 provinces, which have varying areas, ranging 822.7–16,481 km² and with a median of 4621.7 km², and varying populations, from 323,200–8,444,600 and with a median of 1,226,300 people [2]. Dengue follows seasonal patterns in Vietnam with an epidemic period of May–January [3]. The majority of the literature on the determinants of dengue in Vietnam focuses on the short-term effects of climate risk factors, supporting our understanding of the complex environmental conditions that influence dengue occurrence in the country [3,4,5,6,7,8].

However, research on socioeconomic factors influencing human-vector interaction, immunological responses and the likelihood of cases presenting to health authorities in this region has not produced consensus or a clear picture across Vietnam. Schmidt et al., in an individual-level cohort study of 75,000 geo-referenced households in Vietnam over the course of two epidemics, found that villages and peri-urban areas with population density of 3–7000 people/km² were at highest risk of dengue outbreaks [9]. In nearby Thailand, however, Chareonsook et al. found that dengue was in fact more prevalent in rural areas, perhaps due to a proliferation of the less anthropophilic *Aedes albopictus* in these zones [10]. Socioeconomic status also appears to have different associations with dengue risk depending on setting. Wijayanti et al. found high socio-economic status to be a risk factor in urban settings in Java, Indonesia, whilst Qi et al. found that poorer groups in urban settings were at highest risk of contracting dengue in the Pearl River Delta, China [11,12].

Similarly, evidence on spatiotemporal patterns and trends is limited, with a focus on relatively small-scale studies for Vietnam: Cuong et al. found significant spatial clustering of serotypes within 500 metres in Ho Chi Minh City, and in another study found that, in Ho Chi Minh City and its surroundings, “biological and ecologic drivers [of dengue infection] operate at a scale of 50–100 km” [13,14]. In neighbouring Thailand, research focusing on spatiotemporal drivers at a province level have returned promising results: Lauer et al. have produced a spatiotemporal model based on pre-season incidence at a province level that outperforms the 10-year median for 65% of province-level annual forecasts, reduces the mean absolute error by 19%, and successfully forecasts outbreaks over the testing period (2010–2014) [15].

This analysis addresses some of these gaps in the literature on dengue in Vietnam, with the following study aims:

(1) To explore the nature and extent of provincial spatial autocorrelation for dengue case rates in Vietnam;

(2) To determine the association between socioeconomic and spatiotemporal variables and dengue case counts at the province level in Vietnam.

The period of interest is 2013–2015, owing to availability of socioeconomic data. Initial exploration of spatial autocorrelation was completed for the years 2011–2015 in order to allow extraction of spatiotemporal variables with 0–2 year lags for the period of interest 2013–2015. This is in keeping with existing literature that suggests a maximum two-year lag is appropriate for dengue [3].

## 2. Materials and Methods

### 2.1. Data Sources

Data for dengue case counts by province and year were obtained from the National Dengue Control Programme [16]. The programme has run since 1999 and includes all clinical cases and deaths reported by a country-wide provincial public health prevention surveillance network across Vietnam on a monthly and annual basis. The database provides raw case counts by month and year, alongside a population-adjusted annual rate of counts per 100,000 people. Case definition used in reporting follows WHO guidance for provisional diagnosis: acute febrile illness of >38 °C lasting 2–7 days with at least two of the main symptoms including severe headache, retro-orbital pain, nausea, vomiting, myalgia, arthralgia, haemorrhagic manifestations, and leukopenia [16]. In the period 1999–2013, 10–20% of these records were confirmed using serological tests [17]. There is no person identifiable data within this dataset.

Province boundaries were obtained from the Global Administrative Database version 3.6 [18]. Population counts were obtained from the housing census and mid-term population and housing surveys, and publicly available socioeconomic data was downloaded from the General Statistics Office, Vietnam [19]. All but two were used in their original form as downloaded.

(1) Housing pressure: data on total population per housing area was not available, and so a ratio of ‘new’ people to ‘new’ housing was constructed using two datasets:
  inter-annual change in provincial population (numerator);
  square metres of new housing built (denominator).
(2) Data on the percentage of households accessing electricity was only available in two-year intervals, so the mean of the year before and after was taken for inter-year values.

There were no missing data.

### 2.2. Study Design

There are two main sections of analysis in this paper: first, spatial autocorrelation and cluster analysis to determine the extent of spatial autocorrelation in dengue case rates between provinces; second, a regression analysis to explore the association between dengue case counts and spatiotemporal and socioeconomic variables at the province-year level during 2013–2015. The first informs the second; that is, exploration of spatial autocorrelation across multiple years guides the extraction of spatiotemporal variables for the regression analysis. As such, a longer timeframe is used for the first section of analysis, which analysis the period 2011–2015 to provide a lag of up to two years in spatiotemporal variables extracted. A maximum lag of two years was selected because this was the longest period of temporal association found in the literature [3].

#### 2.2.1. Spatial Autocorrelation and Cluster Analysis

To explore spatial autocorrelation for dengue case rates in Vietnam from 2011–2015, two analytical processes were undertaken. First, to determine the overall extent of spatial autocorrelation, a global Moran’s I statistic was calculated in GeoDa version 1.14 (Center for Spatial Data Science (CSDS), University of Chicago, Chicago, United States). The global Moran’s I statistic compares provincial dengue rates with a global expected value across the study area to give an average value of spatial autocorrelation running −1 (indicating dispersion) to +1 (indicating clustering). Its value indicates the extent of clustering overall across Vietnam during the period of interest. Dengue incidence was joined to province boundaries in ArcGIS version 10.3 on a WGS 1984 Web Mercator projection, using geolocation data from the Global Administrative Database version 3.6 [18].

Second, a local Moran’s I statistic was calculated (using Geoda v1.14, (Center for Spatial Data Science (CSDS), University of Chicago, Chicago, United States) to reveal clusters of high or low dengue incidence and outlier provinces (high incidence among low-incidence neighbours, or vice versa). The local Moran’s I statistic uses a weights matrix to compare dengue rates in a given province with a particular set of neighbours and determine significant similarities across these sets. If significant association is found, the sets are marked as clusters (which may be low-low or high-high). Similarly, this statistic marks outlier provinces showing low rates among high rate neighbours, or high among low. Significance for both global and local spatial autocorrelation tests was determined using a Monte Carlo randomisation test of the z-score with 9999 permutations, which produces pseudo *p*-values. Significant clusters were then displayed on maps.

Neighbour sets chosen were first and second order queen contiguous neighbouring provinces. Queen contiguity was chosen over rook in order to capture all bordering neighbours, and contiguity was chosen over distance between incident cases because the data is available by province rather than point location and so a distance-determined assessment of ‘nearby’ incidence, which would require use of a centroid for each province, would pull peripheral cases into the centre of each province and so introduce difference in assessment of clustering dependent on province size. First and second order contiguity were tested because the scale of local biological and ecologic drivers of dengue (estimated at 50–100 km in some areas of Vietnam) may extend beyond first order contiguous neighbours [13,14]. For example, Da Nang province is only 55.2 km east–west and 33.4 km north–south. Third order contiguity was not tested because many provinces in Vietnam have few third order contiguous neighbours, due to its shape. Vietnam has some small islands, for example off the south-west coast of Kien Giang, which are included in provincial counts. Their populations are very small and so not expected to impact analysis outputs; however, this is worth consideration in extrapolating findings for spatial autocorrelation between neighbouring provinces across the entirety of provincial landmass.

#### 2.2.2. Regression Analysis

##### Model

To determine the association between spatiotemporal and socioeconomic variables and dengue case counts at the province-year level in Vietnam from 2013–2015, a negative binomial regression was performed using Stata v15.0 (Statacorp, Texas, United States). Descriptive analysis showed that the outcome variable is over-dispersed count data. The high variance of the data and frequency of zero counts make the Poisson distribution inappropriate. Therefore, negative binomial regression was selected for this model.

Negative binomial regression predicts the log of the outcome with a linear combination of exposure variables:

*log*(*dengue case counts*) = *intercept* + *b*_1_(*var*_1_) + *b*_2_(*var*_2_)…*b_i_*(*var_i_*)

In the above model, intercept is the constant term and *b*_1_, *b*_2_…*b_i_* are the regression coefficients given for each variable of interest.

The above regression equation could also be expressed as

*dengue case counts* = *exp*(*intercept* + *b*_1_(*var*_1_)…+ *b_i_*(*var_i_*))

or

*dengue case counts* = *exp*(*intercept*) × *exp*(*b*_1_(*var*_1_)…× *exp*(*b_i_*(*var_i_*))

As provinces have varying population sizes, provincial population was included as an offset variable after transformation to its natural log (i.e., assuming a multiplicative relationship between population rise and dengue count rise). The average population per province across the period 2011–2015 was used. A year-by-year population figure can be used, however population count varies very little—a maximum change of 0.17% and a mean of 0.01% over the five-year period 2011–2015—so this average is considered an acceptable representation of population across the period of interest.

##### Variables

The outcome variable was dengue case counts by province-year. Socioeconomic variables of interest were

- Factors likely to influence opportunities for human-vector contact: housing pressure (‘new’ people per new m² housing per annum), the percentage of provincial populations living in urban areas [19], population density (people per km²), and population mobility (million-person-km travelled each year) [20];
- Indicators of socioeconomic status: percentage of households accessing electricity and poverty rate (percentage of people with household income below the province-adjusted poverty line) [21];
- Indicators of healthcare access: clinicians per 1000 people and percentage of children under one year vaccinated. Note: vaccination rate is not an indicator for dengue vaccination as this was not available during the period of interest but is instead used a proxy indicator for healthcare access overall;
- A number of other socioeconomic variables, such as age profile and water access, were considered but data was unfortunately not available for this period. Definitions, units, rationale, and source of socioeconomic exposures are in supplementary materials.

Spatiotemporal variables of interest were

- A province’s own case count the previous year;
- A province’s own case count two years previous;
- A province’s first and second order queen contiguous neighbours’ average case count the previous year;
- A province’s first and second order queen contiguous neighbours’ average case count two years previous.

Spatiotemporal variables with a lag of 0–2 years were extracted using Geoda v1.14 (Center for Spatial Data Science (CSDS), University of Chicago, Chicago, United States); for example, the average dengue rates of first order contiguous provincial neighbours the previous year.

A fixed-effects model was used when handling spatiotemporal variables only in the initial univariate regressions, to control for time-invariant and spatially fixed exposures such as altitude. Other known risk factors for dengue, such as rainfall and temperature, do exhibit some inter-annual variation, and so an argument could be made for their inclusion; however, the decision was made that inter-annual variation is not expected to be marked during a three-year period, and it was preferable to exclude them in order to build a parsimonious model. A Hausman test was performed to determine whether fixed or random effects models were more appropriate for socioeconomic variables (results available in supplementary materials). Clinician density, and the proportion of vaccinated infants below one year did not meet the asymptotic assumptions of the Hausman test, and so both of them were brought into both random- and fixed-effects regression models to assess the relative strength of each model. Confounding was considered across available socioeconomic variables. None satisfied the logic of a priori suspected confounders, and so further exploration of confounding (for example, with the Mantel–Haenszel method) was not pursued.

##### Model Development and Testing Goodness of Fit

Regression models were built additively: variables without evidence of a significant association with the outcome (i.e., returning a z or *t* test *p*-value greater than 0.05) were removed at each stage, and models were tested with and without interaction terms. Potential collinearity was explored by constructing correlation coefficient matrices for both spatiotemporal and socioeconomic exposure variables. Where any two variables had a correlation coefficient of 0.5 or more, the variable whose univariate model had the lowest Bayesian Information Criterion (BIC) number was used in subsequent bivariate models. The Bayesian Information Criterion (BIC) number provides an estimate of the posterior probability of a model’s accuracy, with a lower number indicating a model is closer to the ‘true’ model. What constitutes ‘lower’ may be subjective to a particular study and therefore, alongside this, residuals are viewed to test goodness of fit for regression models. Goodness of fit and heteroscedasticity were explored further with raw residual plots (see Supplementary Figures). As there is no assumption of constant variance in negative binomial regression, heteroscedasticity was not formally tested but instead explored using residual plots. Finally, Pregibon’s statistic was calculated to determine goodness of link function (for negative binomial regression, a logarithmic link function) in regression models. Pregibon’s test statistic takes candidate model predictions of the outcome (and their squares) as predictors in a secondary regression. If the link function is correct, the resulting *t* test *p* value should be insignificant.

## 3. Results

### 3.1. Distribution of Dengue Case Counts

**Figure 1:**
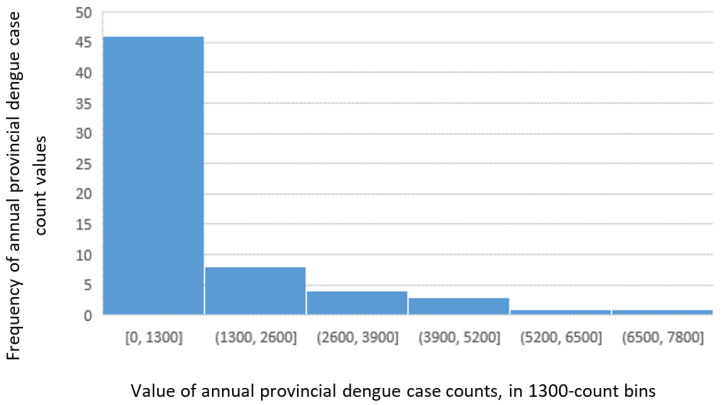
Histogram of dengue case counts per province-year in Vietnam, 2013–2015.

An initial exploration of the distribution of dengue case counts across Vietnam from 2013–2015 shows a strong positive skew (Figure 1), with 15% (=y/n) of provinces showing zero counts in one of the years 2013/2014/2015.The following five provinces had no dengue cases recorded throughout 2013–2015: Bac Kan, Lai Chau, Ha Giang, Lao Cai, and Son La (all highland provinces in the northwest).

### 3.2. Spatiotemporal Trends, Clusters and Outliers

**Figure 2:**
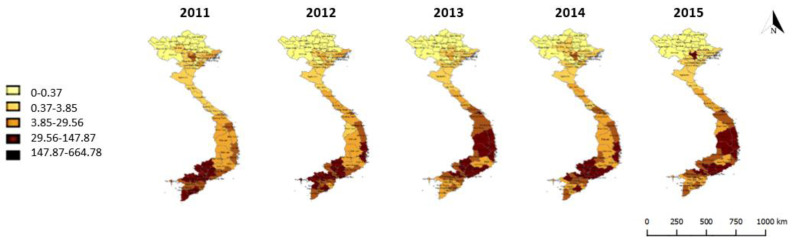
Map of observed dengue cases per 100,000 population, provincial rates, 2011–2015.

Surveying spatial trends in dengue case counts from 2011–2015, the coefficient of variation for annual dengue case counts by province (1.60, Table 1) is greater than 1, and so indicates high spatiotemporal variation in annual dengue case counts. There is a clear north–south trend for dengue counts per 100,000 people, with the exception of Hanoi and some surrounding provinces in the north and shifting high incidence provinces across the southern and central provinces (Figure 2).

Analysis shows that dengue case counts are spatially autocorrelated at the province level across these years—that is, provinces are likely to show similar density of case counts to their neighbours. This is evidenced by Moran’s I statistic values ranging 0.38–0.58 (0 indicating random spatial distribution and 1 indicating extremely high spatial autocorrelation). Monte Carlo simulations produced pseudo *p*-values ranging from 0.00008 (2015) to 0.00001 (2011, 2012, 2014), indicating strong evidence for spatial autocorrelation.

**Figure 3:**
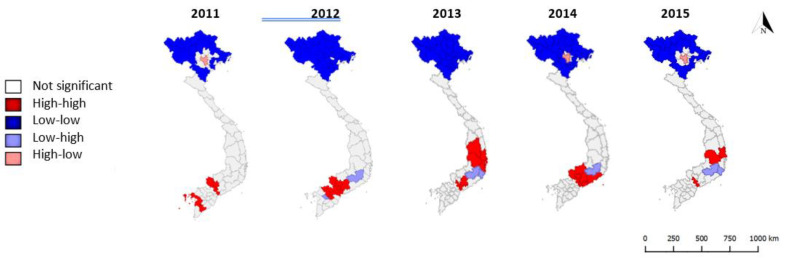
High-high and low-low clusters and outliers (high amongst low or low amongst high) of dengue cases per 100,000 population, first order queen contiguity, 2011–2015.

**Figure 4:**
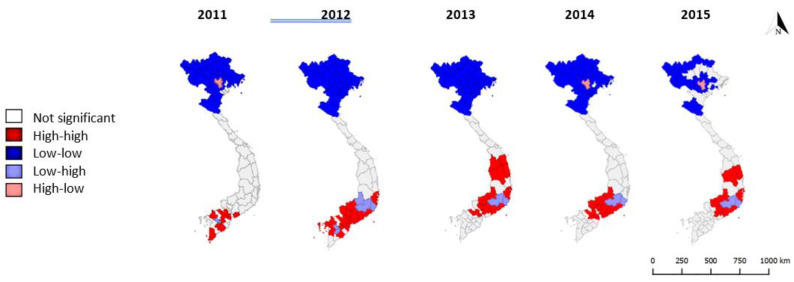
High-high and low-low clusters and outliers (high amongst low or low amongst high) of dengue cases per 100,000 population, second order queen contiguity, 2011–2015.

Clustering is evident across the period 2011–2015, with local Moran’s I statistics indicating clusters of provinces with low and high dengue rates in the north and south respectively for neighbours at both first and second order contiguity (Figure 3 and Figure 4). These trends are in keeping with climatic factors influencing vector capability, with clusters of provinces with low dengue rates in the colder highlands of the north and clusters of provinces with high dengue rates in the tropical lowlands of the south.

Hanoi is a repeated (but not constant) outlier province, with significantly higher rates than its first order contiguous neighbours in three out of five years. Lam Dong province (south/central) is also a repeated outlier, with significantly lower rates than its first order contiguous neighbours in four out of five years. This may suggest population immune response factors at play, or alternative unknown changes in risk factors (such as the impact of El Niño from 2014–2016) [22]. Hotspots (i.e., clusters of high dengue rates) in the south and central regions change annually, with some potential two-year cycles from 2012–2014 and 2013–2015 in some south/central provinces. Cold spots (clusters of low dengue rates) in the north cover the highlands and border provinces consistently, with some variation in the northern inner provinces: provinces immediately surrounding Hanoi do not appear as cold spots in 2011 or 2015.

### 3.3. Regression Analysis

A regression analysis of spatiotemporal variables against dengue case counts produced four models (Table 2) with significant z or *t*-test *p*-values (i.e., <0.05), and very small but significant regression coefficients:

Univariate fixed-effects model with mobility as exposure variable, which showed that an increase of one million-person-km travelled indicates a decrease of one case in the year of interest (*p* = 0.02);

1. Bivariate fixed-effects model with mobility and average dengue rates in first order contiguous neighbours two years previous, which showed that an increase of one million-person-km travelled indicates a decrease of one case in the year of interest (*p* = 0.022), and an increase of one case per 100,000 population averaged across first order contiguous neighbours two years previous indicates an increase of one case in the year of interest (*p* = 0.038). A two-year lag was selected on the basis of previous research indicating a two-year cycle of dengue prevalence may be present [3];
2. A bivariate fixed-effects model with mobility and average dengue rates in second order contiguous neighbours two years previous, which showed that an increase of one million-person-km travelled indicates a decrease of one case in the year of interest (*p* = 0.033), and an increase of one case per 100,000 population averaged across second order contiguous neighbours two years previous indicates an increase of one case in the year of interest (*p* = 0.05);
3. A univariate model with interacting terms taking a multiplication of mobility with dengue rates in the province of interest the previous year, which showed that an increase of one unit of (million-person-km travelled multiplied by dengue rates in the province of interest the previous year) indicates a decrease of one case in the year of interest (*p* = 0.017).

Bringing mobility and dengue rates in the province of interest the previous year together brought down the significance of dengue rates in the province of interest the previous year. Bringing together mobility and dengue rates in both first and second order contiguous neighbours two years previous in a bivariate model, however, returned significant associations and a far lower BIC number than univariate regression models or other bivariate combinations with these same variables. Creating multiplicative interaction terms dramatically reduced regression coefficients—perhaps due to the log link function in negative binomial regression models. Summary statistics for outcome and exposure variables and results from all potential candidate models are available in supplementary materials.

Population density, meanwhile, held a significant but small association with dengue but returned a high BIC. Mobility showed a comparatively lower BIC and significant association with dengue case counts.

### 3.4. Testing Goodness of Fit and Link Function

The above four candidate models exhibited lower Bayesian Information Criterion (BIC) numbers than other uni-, bi-, and tri-variate regression models explored in this analysis. However, BIC numbers were still high at around 1420, and raw residuals exhibited heteroscedasticity: raw residuals tended to be lower where actual case counts were low and extremely high in province-years of high dengue incidence (see Supplementary Materials). Finally, Pregibon’s test returned significant *p*-values for all models, suggesting poor goodness of link in the model (Table 2). Such results suggest a fundamental error in candidate models.

## 4. Discussion

### 4.1. Discussion of Findings

This analysis has yielded three key findings. First, clustering of dengue case rates is evident in both the north and the south of Vietnam, with shifting high-rate clusters across a number of southern provinces that appears to follow a two-year pattern of high incidence. Results of cluster analyses at the provincial level need to be carefully interpreted, as the spatial scale they adhere to is large (see Limitations, below). Indeed, Thai et al. found clustering of cases at the household level in two southern Vietnamese villages [23].

Second, small but significant associations are observed between dengue case counts and mobility, population density, dengue rates the previous year (same province), and dengue rates two years previous (first and second order contiguous neighbours). The association between dengue and both population density and mobility are expected, and the high BIC observed is likely due to the spatial scale of provincial counts being too large to pick up a potential driver of dengue spread at a finer spatial scale. Mobility was included as a potential indicator of multiple serotype circulation and of increasing likelihood of infection through transportation of infected humans and/or vectors, and so a positive association with dengue counts was expected [20]. Such results reinforce the finding of Schmidt et al. that local conditions are complex in determining dengue risk [9]. They may also suggest alternative relationships with dengue, for example high mobility indicating greater economic development and therefore types of employment that discourage human vector contact during biting hours. An exploration of such potential relationship requires, however, a more nuanced and deep understanding of the exposure variables than that available for this analysis. Published data for mobility, for example, does not specify whether more million-person-km represents many short journeys, fewer longer journeys, and indeed of who and using what form of transport—all important pieces of information necessary to draw firm epidemiological conclusions from this regression output. Likewise, there is little detail on the nature of provincial boundaries—which, bordering both provinces, countries and sea mass, may represent markedly different levels of porousness or controls on mobility. Further analysis looking across national boundaries could be instructive here.

Turning to spatiotemporal variables, it was expected that stronger and positive associations would be seen for spatially and temporally closer province-years. Two-year time lags held stronger associations with dengue case counts in the year of interest than one-year time lags. This is supported by existing literature, which indicates epidemic cycles over two years in Southern Vietnam, and may be linked to population immunological cycles, a two-year process of vector movement across provinces, or human movement between provinces introducing either new infection or new serotypes over the course of two epidemic seasons [3,14]. The negative association seen with a province’s own dengue case incidence the previous year may indicate immunological protection for populations in previously high-incident populations, or lack of protection where populations have ‘missed’ an epidemic season. This would only be the case for mono-serotype sequential infection and so could suggest limited serotype circulation across provinces in Vietnam. Wider serotype testing would enable exploration of this hypothesis [24].

Third, significant associations were not found between dengue case counts and the following socioeconomic variables: people per m² housing; access to electricity; clinician density; province-adjusted poverty rate; percentage of under-1s vaccinated; percentage of population in urban settings. The lack of significant association between dengue case counts and socioeconomic variables beyond mobility is unexpected and challenges the literature [21]. The non-association between dengue case counts and clinician density and vaccination rates in infants below one year of age are particularly surprising as case reporting requires strong data and health infrastructure, as well as access to clinicians for those exhibiting symptoms. As such, one would expect a strong and positive relationship between dengue counts and variables indicating health system strength. This may be due to the coarse level of aggregation. Whilst climate variables are homogenous across large spatial areas, socioeconomic indicators are largely heterogeneous. Making them more homogenous across space may reduce the size and significance of association seen.

For all regression outputs, it is important to note that raw residual plots (supplementary materials) and predicted case count values (Table 3) suggest that these models are not appropriate as predictors of dengue case counts at the province level across Vietnam (2013–2015). The purpose of this analysis is to identify which of the candidate variables are associated with the regional pattern of dengue in Vietnam, rather than to explain or predict this pattern or the spatial correlation observed. In order to assess this further, researchers could gather data at a finer spatio-temporal scale and determine the extent to which these variables aid prediction of future incidence and/or explain the variation observed.

### 4.2. Limitations

There are many reasons to be cautious in interpreting these findings, and there are several limitations to this analysis.

#### 4.2.1. Modelling Limitations

There may be issues with the modelling approach selected in this analysis that warrants further investigation using alternative methods. First, the fixed-effects negative binomial regression command in Stata 15.0 automatically excluded provinces with zero counts across all years of interest. This is problematic as it excludes the five highland provinces (listed in results) and may introduce systematic bias and skew results. Therefore, any further research on this topic would do well to consider alternative software packages and/or commands for fixed effects negative binomial regression. In future analyses, a standard negative binomial regression with neither fixed nor random effects specified could also be tested to ensure provinces with zero counts are included. Such a model would require the inclusion of time-invariant known risk factors such as altitude, which was not done in this analysis due to limited time and easily available data. Although a Hausman test is used to determine whether fixed or random effects are more appropriate, it is worth noting that some exposures (such as housing pressure) showed different levels of temporal variation across different provinces, so fixed-effects may be mis-specified. This is further suggested by very low regression coefficients; time-invariant variables return a zero coefficient on fixed-effects negative binomial regression. Further, this study comprises multiple observations at different times coming from the same province, which are likely to be correlated with one another in a form of pseudo-replication. This issue could be addressed by using a random effects model in future analyses and comparing results. None of the candidate models included variables with a high correlation coefficient, and so risk of collinearity is considered low (see the Supplementary Materials for correlation coefficients matrices for both spatiotemporal and socioeconomic exposure variables).

The Pregibon goodness of link test results suggest that the negative binomial log-link function may be mis-specified for this data (Table 3). An alternative approach that enables simultaneous analysis of zero and non-zero count data, such as hurdle models, may be instructive in determining the multiple relationships at play between over-dispersed dengue counts and their potential socioeconomic and spatiotemporal associations [25].

Residuals are markedly high across all final candidate models. This is perhaps unsurprising given the level of dispersion in the data and the appearance of very high case counts in some province-years, apparently at random (see supplementary tables for detail on dispersion, e.g., standard deviation). That aspect of the outcome data is fundamentally challenging to the model, and indicates potential issues with applying a single model to the whole of Vietnam. It may not be appropriate to group all provinces together in a single regression model. This is in part because the outcome varies so much, and a single model requires the ‘flattening’ of those differences. Sub-analyses for similar socioeconomic or climatic regions (for example, the Mekong River Delta) might also return a more sensible estimation of the different relationships at play between dengue case counts and spatiotemporal and socioeconomic variables. This was not undertaken for this analysis due to limited data.

#### 4.2.2. Data Limitations

There are some important caveats to the data used which are important to note during interpretation of results. Above all, readers should keep in mind that this study uses data on reported rather than true cases. Indeed, Cuong et al. calculated a conservative estimate of the specificity of a clinical dengue case diagnosis in Vietnam in 2013 at ≈50%, based on IgM in one serum sample collected from a small proportion (<10%) of patients [26]. The limited specificity of case reporting is itself an issue; there is also a chance that clinician and patient awareness of nearby dengue outbreaks may exacerbate this, and falsely amplify spatial autocorrelation. Clinicians in a hyper-endemic area are more likely to have both training and experience to recognise dengue in generic febrile symptoms, whilst those unused to seeing it may mis-diagnose dengue as another febrile condition. This may be equally so for patients, who may be quicker to present themselves to health services if they are aware of dengue in their area. Spatial autocorrelation, particularly in historically hyper-endemic areas, should therefore be treated with caution. To address this limitation, further analysis of spatial autocorrelation within clusters or in outlier provinces at a more granular spatiotemporal scale may be instructive. Alternative analytical approaches, such as wavelet analysis to determine inter-epidemic temporal lags or development of probabilistic early warning systems—as explored by Lowe et al., in Brazil—could also support predictions of dengue risk in Vietnam [27]. Further investigation might also helpfully explore dengue rates in neighbouring countries, and sub-group analyses for climatic or socioeconomic zones which may determine a cross-national area more epidemiologically important as a footprint for analyses than national borders.

Turning to exposure variables, data has been collected in different ways: mobility, for example, is determined by official estimations based on traffic data; electricity access, meanwhile, is recorded using household surveys. Exposure variables explored are also selected on the basis of available data and do not necessarily represent the only epidemiologically plausible exposures, which may also include (once such data is available) age profiles or employment type [28].

For all variables in this analysis, data quality is poorly understood. There are no indicators of data quality (such as completeness of data returns from each province) published alongside either dengue or socioeconomic datasets. A multinational comparison of dengue case reporting and surveillance systems found Vietnamese surveillance to be comparatively accurate in reporting isolate information from 2004 onward [29]. This observation is encouraging; however, questions remain as to the objective quality and completeness of the dengue surveillance data used for this analysis.

Finally, and perhaps most critically, data is limited. Observations available for this analysis are constrained to the three years for which all socioeconomic variables of interest published, and zero-count outcome provinces (of which there were five) were automatically excluded under the fixed-effects negative binomial regression command in Stata 15.0 (see above). A further analysis is recommended when more years of socioeconomic data are released.

#### 4.2.3. Spatiotemporal Scale Limitations

The potential extent of heterogeneity for both exposures and outcome within such a large spatial scale is worth noting. Some exposures may not overlap with the outcome at all in spite of both being present within a single province, and socioeconomic factors may have different influences on different communities or at different scales. Low electricity access in an urban setting, for example, may be associated with poor housing, overcrowding and, as a consequence, higher risk of infection with dengue. Low electricity access in rural highland areas, meanwhile, may indicate more remote and high-altitude settings and therefore reduced risk of dengue. Indeed, Delmelle et al. found spatial heterogeneity of association between socioeconomic factors and dengue risk at the neighbourhood level in a similar analysis in Cali, Colombia [30].

Beyond this, provinces are not only large, they also have varying geographic areas. This may mean inter-provincial clustering is more readily evident across multiple small provinces and less so across fewer larger provinces, although dengue is still spreading across the same area. It may also mean that some provinces contain different levels of heterogeneity in key risk factors, such as altitude [3,4]. Spatial analysis relying on contiguous provinces of varying sizes does not therefore offer a standardised picture of spatial autocorrelation (such as a proposed spatial scale of dengue spread in Vietnam), but rather a picture of provincial autocorrelation. Just as importantly, the temporal scale of this analysis—a year running 1 January to 31 December—does not correspond to temporal fluctuations in dengue (typically peaking in June–October) and so may not be epidemiologically appropriate, and may miss fluctuations in exposure variables such as mobility. If socioeconomic data becomes available, the authors recommend further analysis at a finer temporal scale.

A further consideration with regards the spatial boundaries employed is the overall geographical layout of Vietnam. It has a long, thin shape which means a high proportion of provinces border either coastline or landmass in other countries which are not included in this analysis. This has implications for spatial analyses considering average rates across neighbours, as some provinces have ‘invisible’ neighbours in, for example, Cambodia.

## 5. Conclusions

In spite of these limitations, this analysis has offered a number of insights as to the spatiotemporal dynamics and potential socioeconomic drivers of dengue spread across large spatiotemporal and population scales. Its findings could support the targeting of prevention efforts, for example, in southern provinces with high dengue case counts following two-year cycles, and open avenues for further research, such as other factors at play in the changing location of outlier provinces over time. Non-association flagged by this analysis has been as important as association and has challenged assumptions about the transmission dynamics and case reporting of dengue in Vietnam.

This analysis has also opened up a number of avenues for further research into dengue that need not be limited to Vietnam, nor to dengue alone. Further exploration of these relationships in other settings and for other arboviruses carried by *Aedes aegypti* and *Aedes albopictus* (including Zika and Chikungunya) would be instructive, as well as looking into their impact on mortality as well as morbidity.

Above all, this study and its limitations demonstrate that the interactions between socioeconomic status, time, space and dengue spread are complex and warrant investigation together and on multiple spatiotemporal scales. We hope it will prompt further exploration of these questions, and a richer understanding of this complex disease.

## Figures and Tables

**Table 1:** Summary statistics, dengue rates (counts per 100,000 population by province-year), 2011–2015.

Annual Dengue Rates, All Provinces, 2011–2015	
Arithmetic mean	66.41
Maximum	731.86
Minimum	0
Standard deviation	106.42
Coefficient of variation	1.60

**Table 2:** Summary of four final candidate model regression outputs (rounded to two significant figures). 174 province-year observations used.

Regression Results						
Model	Wald Chi *p* Value	Coefficient (Exponentiated)		z or *t* Test *p* Value	Constant/Intercept	BIC Number
1	0.02	Mobility	−1.00	0.02	−13.54	1419.83
2	0.0075	Mobility	−1.00	0.022	−13.71	1420.88
		First order neighbours two years previous	1.00	0.038		
3	0.0093	Mobility	−1.00	0.033	−13.73	1421.30
		Second order neighbours two years previous	1.00	0.05		
4	0.017	Mobility	−1.00	0.017	−13.63	1417.95

**Table 3:** Summary of four final candidate model predicted case counts and Pregibon’s statistic.

Model	Predicted vs. Actual Summary Statistics (Provincial Dengue Case Counts, 2013–2015)								Pregibon Test *p* Value
	Min		Max		Mean		Standard Deviation		
	Predicted	Actual	Predicted	Actual	Predicted	Actual	Predicted	Actual	
1	0.2	0	9.39	5610	1.3	43.27	1.7	159.96	0.0006
2	0.25		9.49		1.27		1.72		0.0002
3	0.22		8.5		1.27		1.72		0.0005
4	0.37		9.03		1.3		1.68		0.0001

## Supplementary Materials

The following are available online at https://www.mdpi.com/2414-6366/5/2/81/s1; Figure S1: Moran’s I graphs demonstrating cross-provincial spatial autocorrelation for second order queen contiguous neighbours, 2011–2015. Table S1: Summary statistics, dengue case counts 2013–2015. Table S2: Definitions, units, rationale and source of socioeconomic exposures (annual province-level data for the years 2013–2015 inclusive). Table S3: Vietnam country population summary statistics. Table S4: Summary statistics for spatiotemporal exposure variables 2011–2015 (2.d.p.; see Table 2 for variable descriptions). Table S5: Correlation coefficients matrix, spatiotemporal variables (2.d.p.; see Table 2 for variable descriptions). Table S6: Summary statistics, socioeconomic variables (2.d.p.). Table S7: Correlation coefficients matrix, socioeconomic variables. Table S8: Results of the Hausman test for socioeconomic variables (2.s.f.). Table S9: Pregibon’s test results for candidate models (see Table 2 for spatiotemporal variable descriptions). Table S10. Summary of potential candidate model regression outputs (2.s.f.; see Table 2 for description of spatiotemporal variables).
