# Supplementary material for: Spatiotemporal and Socioeconomic Risk Factors for Dengue at the Province Level in Vietnam, 2013–2015: Clustering Analysis and Regression Model
Source: Trop Med Infect Dis. 2020 May 19;5(2):81. doi: 10.3390/tropicalmed5020081 (PMC7345007; doi:10.3390/tropicalmed5020081)

Supplementary Materials

Supplementary Table S1. Summary statistics, dengue case counts 2013–2015.

**Annual dengue case counts, all province-years 2013-15**	
Arithmetic mean	1117.898
Maximum	15412
Minimum	0
Standard deviation	2046.175
Coefficient of variation	1.83

Supplementary Table S2. Definitions, units, rationale and source of socioeconomic exposures (annual province-level data for the years 2013–2015 inclusive).

Poverty rate	The percentage of people or households with per capita income lower than the poverty line. The poverty line is the cost of a basket of essential food (determined by each province as locally appropriate) plus a minimum level of expenditure for non-food items such as housing, clothing, household appliances, 'cultural learning' items, entertainment, medicine, and travel. It is adjusted by province-weighted consumer price index.	Percent (people), continuous variable	The literature on association between dengue and poverty is conflicted, and there is no country-wide analysis of this for Vietnam. It is therefore an important avenue for consideration in this analysis.	Survey of Vietnamese People's Living Standards<br><br>General Statistics Office of Vietnam
Electricity access	Percentage of households with access to electricity.	Percent (households), continuous variable	Electricity access is an indicator of the quality of public infrastructure in each province, which is relevant for public health planners trying to ascertain its association with dengue fever in the context of	General statistics Office of Vietnam.

			increasingly developed public infrastructure as Vietnam's economy grows.	
Urban population	Percentage of population living in urban areas	Percent (urban people), continuous variable	Multiple studies have indicated association between dengue and urbanisation.	General Statistics Office of Vietnam.
Housing pressure	A metric calculated for this dissertation, using change in total population from previous to current year as numerator, and thousand square metres of new housing built for residential and domestic purposes as denominator. This gives 'new' people per new square metre of housing built.	'New' people per new square metre housing, continuous variable	Greater density of people to housing may create a risk of overcrowded or casual housing. These in turn may have an impact on human-vector contact patterns through increased time outside the home at dawn and dusk, or greater proximity to larval maturation sites – for example in abandoned containers around casual urban or peri-urban settlements.	New housing data from reporting on construction activities and enterprise, through a network at ministerial and branch levels, and published by the General Statistics Office, Vietnam. Population change as below (*population density*).
Population density	Division of provincial population by provincial area.	People per square kilometre, continuous variable	The association between population density and dengue across Vietnam is an open question, with some highly dense areas (Hanoi, Ho Chi Minh City) exhibiting high incidence whilst Schmidt et al found a narrow range of moderate population density was associated with higher dengue incidence in Kanh-Hoa province.	General Statistics Office of Vietnam.
Mobility	The number of person-km travelled in a province, all forms of transport (but dominated by road traffic)	Million-person-km, continuous variable	This metric indicates provincial interconnectedness and population mobility, which in turn may spread epidemics and increase serotype circulation. This in turn increases the risk of more severe forms of dengue warranting hospitalisation and, as a consequence, case detection.	Official surveys of transport, published by the General statistics Office of Vietnam.

Under-1 vaccination	Percentage of children under one year of age vaccinated according to the Vietnamese Ministry of Health vaccine schedule.	Percent (under-1s vaccinated), continuous variable	This is an indicator for healthcare access and community engagement with the health system. The vaccine schedule for 2013–2015 does not include dengue vaccination.	Demographic and Health Survey reported by the Ministry of Health via the General Statistics Office of Vietnam.
Clinician density	A metric calculated for this dissertation. All doctors, clinical assistants and nurses per province-year, as listed in official statistics on health workforce, adjusted to a per-thousand-people clinician density metric using population counts (Table 4). This excludes midwives as they are not expected to support treatment or case definition of dengue fever.	Clinicians per 1000 people, continuous variable	As the outcome is cases reported rather than true cases, and clinicians are the agents reporting cases, clinician density is expected to be positively associated with dengue case counts. It is also an important indicator of healthcare access. Clinicians per thousand people was selected over bed or clinic numbers as it encompasses a broader range of treatment settings (such as home visits or outpatient clinics).	Investigations of human resources for health workforce, published by General Statistics Office of Vietnam.

Supplementary Table S3. Vietnam country population summary statistics.

	% change annual provincial population count between two consecutive years during the period 2011-15	% change annual provincial population count between the first and last years of 2011-15	Average population count by province across the 5-year period 2011-15
Min	0.00028	0.001848	305800
Max	0.043337	0.173648	7821380
Mean	0.009944	0.042165	1506176
Standard Deviation	0.00765	0.0318	1,242,717

Supplementary Table S4. Summary statistics for spatiotemporal exposure variables 2011–2015 (2.d.p.; see Table 2 for variable descriptions).

Exposure variable (all dengue counts per 100,000 people)	Observations	Mean	Standard deviation	Min	Max
Y1own	315	1166.94	2062.21	0	18872.72
Y2own	315	1386.78	2313.91	0	18872.72
Y1q1	315	1177.10	1520.43	0	10968
Y1q2	315	1194.92	1416.97	1	9436.5
Y2q1	315	1421.13	1597.78	0	10968
Y2q2	315	1453.09	1474.46	1	9436.5

Supplementary Table S5. Correlation coefficients matrix, spatiotemporal variables (2.d.p.; see table 2 for variable descriptions).

	Y1own	Y2own	Y1q1	Y1q2	Y2q1	Y2q2
Y1own	1					
Y2own	0.60	1				
Y1q1	0.68	0.45	1			
Y1q2	0.66	0.67	0.88	1		
Y2q1	0.46	0.67	0.66	0.62	1	
Y2q2	0.48	0.62	0.61	0.68	0.85	1

Supplementary Table S6. Summary statistics, socioeconomic variables (2.d.p.).

Variable	Obs	Mean (2.d.p.)	Std. Dev.	Min	Max
Poverty rate (%)	189	11.59	8.38	0	41
Population density (people per km^2)	189	481.35	588.58	45	3888
Clinician density (clinicians per thousand people)	189	3.13	2.48	1	22
Mobility (million-person-km travelled, all forms of transport)	189	1567.27	2396.80	7	17194
Housing pressure ('new' people per square metre new housing built)	189	8.45	17.86	1	139
Under-1 vaccination rate (%)	189	95.61	5.05	74	100

Electricity access (households accessing electricity, %)	189	97.25	5.83	65	100
Urban population (%)	189	28.25	17.01	10	88

Supplementary Table S7. Correlation coefficients matrix, socioeconomic variables.

	**Povert y rate**	**Pop. density**	**Clin. density**	**Mobility**	**Housing pressure**	**Under 1 vacc. rate**	**Access to elec.**	**Urban popula tion**
Poverty rate	1							
Population density	-0.4562	1						
Clinician density	0.0582	-0.1742	1					
Mobility	-0.4099	0.2102	0.5759	1				
Housing pressure	-0.0052	0.5435	-0.1336	-0.0464	1			
Under 1 vaccination rate	-0.2988	0.1604	-0.0563	0.0384	0.0481	1		
Access to electricity	-0.4836	0.1671	-0.1	0.0888	-0.0402	0.1492	1	
Urban population	-0.3839	0.4355	-0.1925	0.1414	0.4483	0.0453	-0.0875	1

Supplementary Table S8. Results of the Hausman test for socioeconomic variables (2.s.f.).

Variable	Hausman P value (if <0.05, fixed effects more appropriate)	Appropriate model
Poverty	0.0004	Fixed effects
Mobility	0.042	Fixed effects
Access to electricity	<0.0001	Fixed effects
Urban population	<0.0001	Fixed effects
Housing pressure	0.55	Random effects
Population density	0.08	Random effects

Supplementary Table S9. Pregibon's test results for candidate models (see Table 2 for spatiotemporal variable descriptions).

	Candidate model	Pregibon test P value
**1**	Mobility	0.0006
2	Mobility + y2q1	0.0002
3	Mobility + y2q2	0.0005
4	Mobility * y1own	0.0001

Supplementary Table S10. Summary of potential candidate model regression outputs (2.s.f.; see Table 2 for description of spatiotemporal variables).

	Variable	Fixed or random effects	Observations	Wald chi square statistic p value	Coefficient	Two-tailed P value for coefficient z or t test	Constant/ intercept	Bayesian information criterion (if two-tailed z test p value <0.05)
Potential candidate	Y1own + y2q1	Fixed	174	<0.0001	Y1own -0.000080<br>Y2q1 0.000076	<0.001<br>0.011	-13.82	2857.30
	Y1own + y2q2			<0.0001	Y1own -0.000078<br>Y2q2 0.0001011	<0.001<br>0.002	-13.86	2854.92
	Y1own		285[1]	<0.001	-0.000088	<0.001	-13.69	2857.29
	Y2q1			<0.001	0.00010	<0.001	-14.074	2872.44
	Y2q2			<0.001	0.00013	<0.001	-14.11	2868.70
	Population density	Random	189	0.04	-0.00036	0.04	-13.57	2506.15

[1] 30 observations (total n=315) dropped due to all zero outcomes

Tropical Medicine and Infectious Disease

MDPI

Annex B: Full results of uni-, bi-, and tri-variate regressions with and without interaction terms. Values are rounded to two significant figures; see Table 2 for spatiotemporal variable descriptions. Preliminary candidate models are marked in light green; final four candidate models are marked in dark green.

Univariate models						
**Spatiotemporal exposures for 2011–2015**						
**Variable**	**Fixed or random effects**	**Observations**	**Coefficient**	**Two-tailed P value for coefficient z test**	**Constant/ intercept**	**Bayesian information criterion (if two-tailed z test p value <0.05)**
Y1own	Fixed	N=285 (6 provinces (30 obs) dropped because of all zero outcomes.)	-0.000088	<0.001	-13.69	2857.29
Y2q1			0.00010	<0.001	-14.074	2872.44
Y2q2			0.00013	<0.001	-14.11	2868.70
Y2own			0.0000040	0.80	-13.92	N/A
Y1q1			-0.000055	0.10	-13.83	N/A
Y1q2			-0.000021	0.59	-13.89	N/A
**Socioeconomic exposures for 2013–2015**						
**Variable**	**Fixed or random effects**	**Observations**	**Coefficient**	**Two-tailed P value for coefficient z test**	**Constant/ intercept**	**Bayesian information criterion (if two-tailed z test p value <0.05)**
Poverty	Fixed	N=174 (5 provinces dropped due to all zero outcomes)	-0.026	0.21	-13.57	N/A
Mobility			-0.00011	0.02	-13.54	1419.83
Electricity			-0.044	0.29	-9.45	N/A
Urban			-0.0032	0.65	-13.72	N/A
Population density	Random	N=189 (no provinces dropped)	-0.00036	0.04	-13.57	2506.15
Housing pressure			0.00028	0.97	-13.85	N/A
Clinician density	Fixed	174	0.0061	0.87	-13.84	
	Random	189	-0.019	0.68	-13.80	
Under 1 vaccination rate	Fixed	174	-0.0089	0.56	-12.96	
	Random	189	-0.0010	0.95	-13.75	

Bivariate models							
**Exposure**	**Obs**	**Wald chi squared statistic P value**	**Coefficients**		**Two-tailed P value for coefficient z test**	**Intercept**	**BIC number**
Y1own + y2q1	174	<0.0001	Y1own	-0.000080	<0.001	-13.82	2857.30
			Y2q1	0.000076	0.011		
Y1own + y2q2		<0.0001	Y1own	-0.000078	<0.001	-13.86	2854.92

Y2q1 + y2q2		0.0001	Y2q2<br>Y2q1<br>Y2q2	0.0001011<br>0.00011<br>0.000063	0.002<br>0.005<br>0.071	-14.16	N/A
Mobility + y1own		0.027	Mobility<br>Y1own	-0.00010<br>-0.000066	0.032<br>0.18	-13.46	N/A
Mobility + y2q1		0.0075	Mobility<br>Y2q1	-0.00011<br>0.00014	0.022<br>0.038	-13.71	1420.88
Mobility + y2q2		0.0093	Mobility<br>Y2q2	-0.00010<br>0.00014	0.033<br>0.05	-13.73	1421.30
**With interaction terms**							
Y1own * y2q1		0.98	0.00000000056		0.98	-13.82	N/A
Y1own * y2q2		0.64	-0.000000010		0.64	-13.80	N/A
Y2q1 * y2q2	174	0.29	0.000000025		0.29	-13.88	N/A
Mobility * y1own		0.017	-0.000000041		0.017	-13.63	1417.95

**Trivariate models**						
**Variables**	**Wald chi squared statistic**	**Regression coefficient value**		**Two-tailed P value for coefficient z test**	**Constant/ intercept**	**Bayesian information criterion (if two-tailed z test p value <0.05 for both exposure variables)**
Y1own + y2q1 _ y2q2	<0.0001	Y1own<br>Y2q1<br>Y2q2	-0.000075<br>0.000081<br>0.000045	<0.001<br>0.03<br>0.20	-13.90	N/A
Mobility + y1own + y2q1	0.0069	Mobility<br>Y1own<br>Y2q1	-0.00010<br>-0.000074<br>0.00014	0.03<br>0.14<br>0.029	-13.62	N/A
Mobility + y1own + y2q2	0.0057	Mobility<br>Y1own<br>Y2q2	-0.000092<br>-0.000086<br>0.00016	0.055<br>0.092<br>0.022	-13.65	N/A
Mobility + y2q1 + y2q2	0.017	Mobility<br>Y2q1<br>Y2q2	-0.00011<br>0.000068<br>0.000092	0.027<br>0.52<br>0.36	-13.75	N/A
**With interaction terms**						
Y1own * y2q1 * y2q2	0.83	-0.0000000000022		0.83	-13.81	N/A
Y1own * Mobility * y2q1	0.91	-0.00000000000091		0.91	-13.82	N/A
Y1own * Mobility * y2q2	0.70	-0.0000000000039		0.70	-13.80	N/A

Mobility * y2q1 * y2q2	0.84	-0.00000000000092	0.84	-13.81	N/A

Supplementary Figure S1. Moran's I graphs demonstrating cross-provincial spatial autocorrelation for second order queen contiguous neighbours, 2011–2015.

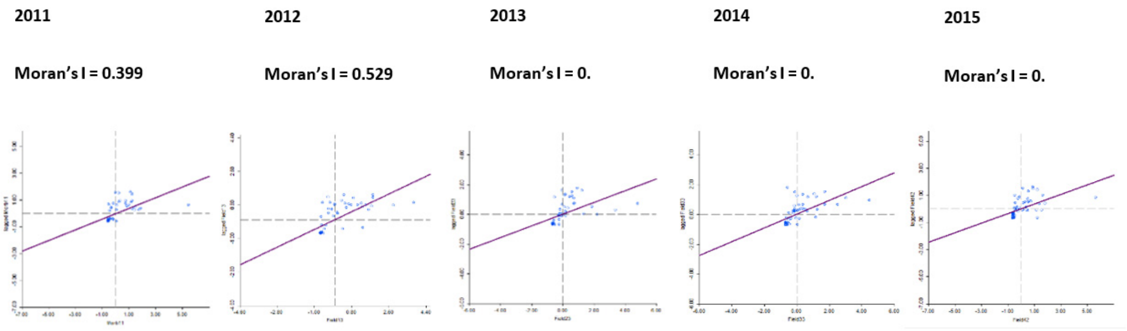

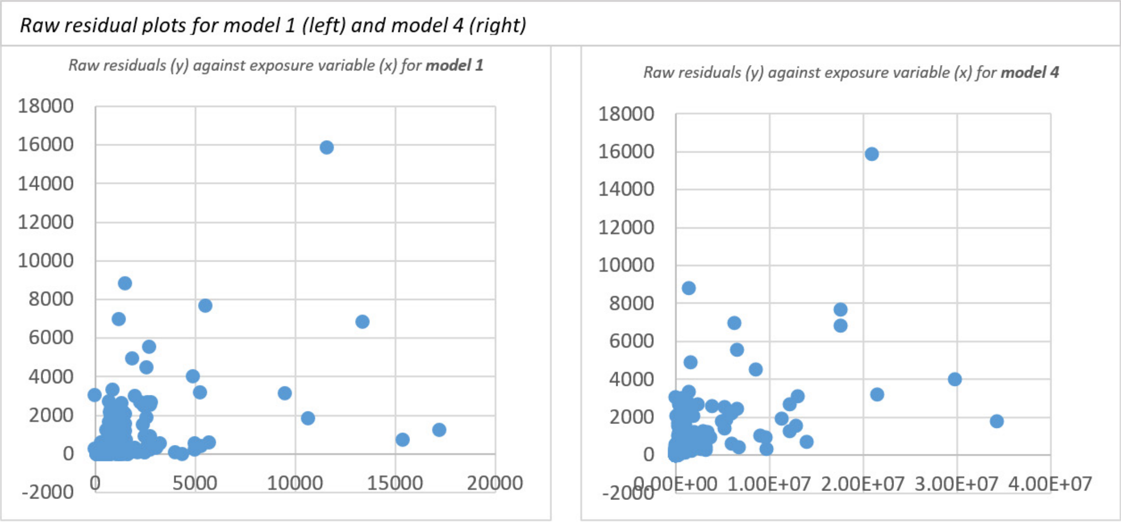

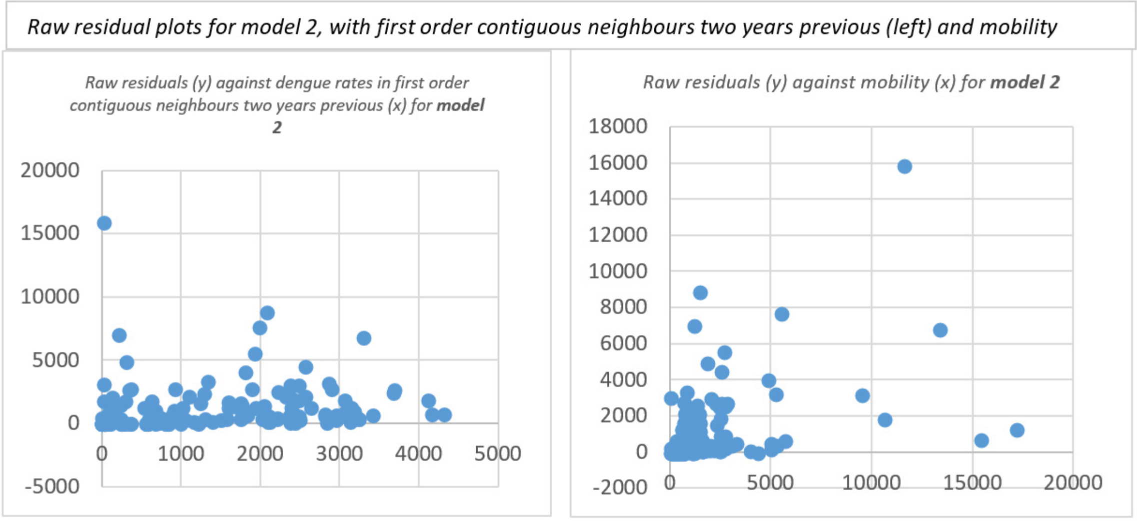

Raw residual plots for model 3, with second order contiguous neighbours two years previous (left) and mobility

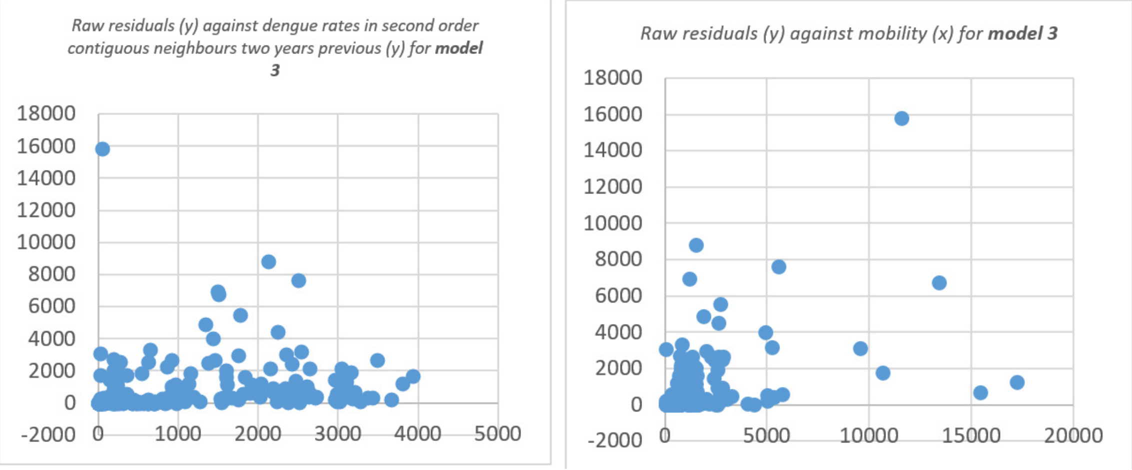

Raw residuals plot for log-transformed mobility univariate model (model 1, log-transformed)

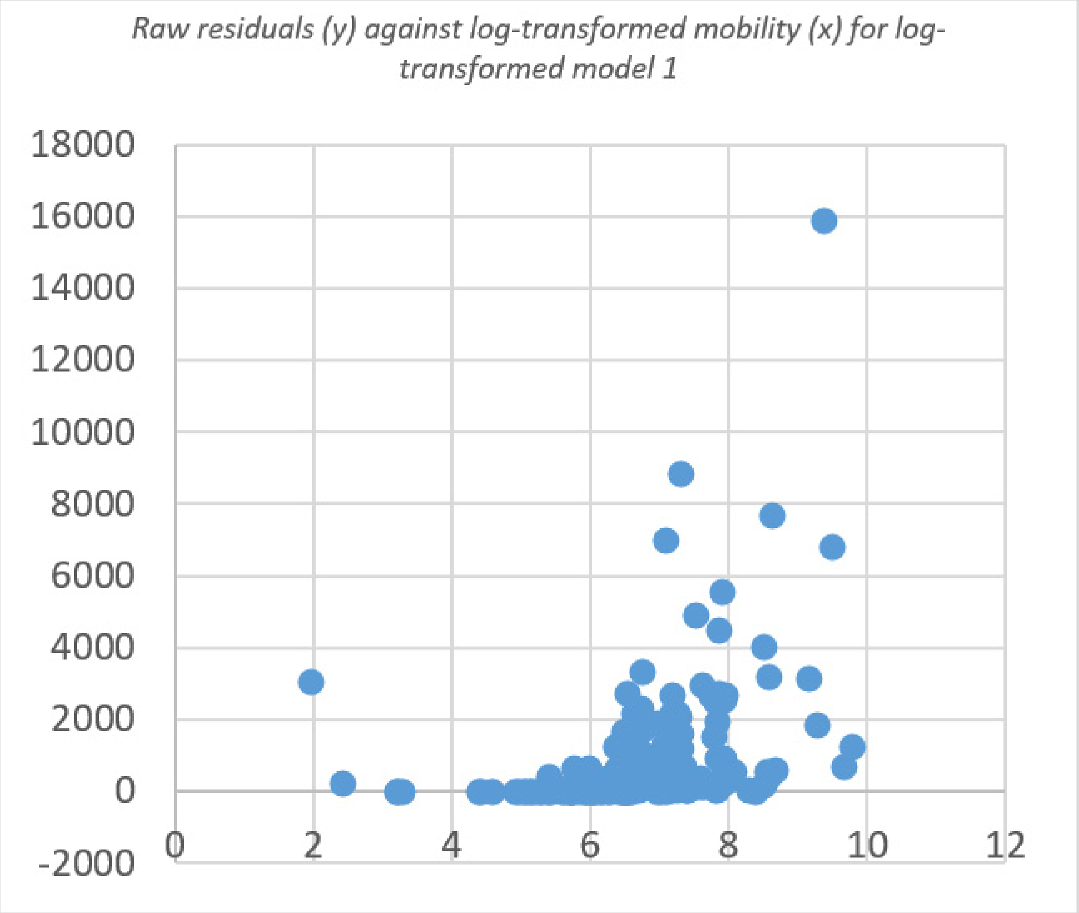

Supplement: Supplementary file 1 [file tropicalmed-05-00081-s001.pdf]
